# Comparison of Clinical and Pathological T Staging in Gingivobuccal Complex Cancers—A Retrospective Analysis

**DOI:** 10.1007/s13193-025-02333-5

**Published:** 2025-06-02

**Authors:** Poonam Joshi, Sameema V. V., Rathan Shetty K. S., Arjun Singh, Asawari Patil, Sudhir V. Nair, Pankaj Chaturvedi

**Affiliations:** 1 Head and Neck Surgical Oncology, ACTREC – TMC, Navi Mumbai, India; 2 Department of Pathology, TMH, Mumbai, India

**Keywords:** Clinical T staging, Pathological T staging, Gingivobuccal complex (GBC) cancers

## Abstract

Accurate staging of gingivobuccal complex (GBC) cancers is critical for treatment planning and prognosis. Discrepancies between clinical T staging (cT) and pathological T staging (pT) may lead to over or undertreatment. This study examines these discrepancies to highlight their implications. A retrospective observational study of 469 patients with GBC cancers was conducted at a tertiary care center. Patients underwent clinical staging based on imaging and physical examination and pathological staging following surgical resection. The agreement between cT and pT stages was analyzed using Cohen’s kappa coefficient. Concordance between cT and pT stages was 32.08%. Upstaging (pT > cT) occurred in 13.4% of cases, primarily due to extra nodal extension or nodal metastases, while 51.4% of cases were downstaged, often from cT4b to pT4a. Discrepancies arose from misjudgements of tumor size, depth of invasion, and involvement of structures like the masticator space, infratemporal fossa, and bone. Significant discrepancies between cT and pT staging in GBC cancers underscore the limitations of preoperative assessments. Integrating advanced imaging and tagging structures intraoperatively could improve staging accuracy, optimize treatment decisions, and enhance outcomes.

## Introduction

Gingivobuccal complex cancers involving buccal mucosa, alveolar ridge, and gingivobuccal sulcus is the most common site for oral cancers among Indian population. These cancers are particularly prevalent among the people who consumes tobacco and betel quid in different forms. They are often associated with late-stage presentation, aggressive behavior, and poor prognosis.

Accurate staging of GBC cancers is essential for determining appropriate treatment and predicting patient outcomes. Eighth edition of AJCC staging system is most used one, which is based on both clinical and pathological evaluation. The T stage, which refers to the size and extent of the primary tumor, plays a crucial role in guiding therapeutic strategies. However, discrepancies between clinical T staging (cT) and pathological T staging (pT) can influence prognosis, treatment decisions, and overall survival of the patients. These discrepancies can lead to either upstaging or downstaging, which can further cause either undertreatment or overtreatment of the patients. The factors that can influence the accuracy of T staging are size, tumor thickness, bone erosion, skin involvement, and masticator space and infratemporal fossa involvement. As per literature, staging discrepancies is found in around 50% of the head and neck cancer patients [1]. Our study aims to compare clinical and pathological T staging in gingivobuccal complex cancers, highlighting the importance of precise staging techniques and the implications of discrepancies in staging system.

## Materials and Methods

This is a retrospective observational study aimed at comparing the clinical and pathological T staging of patients diagnosed with gingivobuccal complex cancers. The study was conducted at ACTREC, Tata Memorial Centre, Navi Mumbai. The study included patients diagnosed with squamous cell carcinoma of the gingivobuccal complex who underwent primary surgical resection between January 2022 and December 2023. Only patients with both preoperative clinical T staging and postoperative pathological staging available were included. Patients with incomplete records, previous history of head and neck cancers, negative histology, or those who received neoadjuvant therapy prior to surgery were excluded from the study. Patient demographics, clinical history, and treatment details were extracted from electronic medical records.

The clinical T staging was determined by physical examination and imaging studies, including CT or MRI, based on AJCC (American Joint Committee on Cancer) 8 th edition. Pathological T staging was determined postoperatively based on the histopathological examination of the surgical specimens by experienced pathologists, following the same staging criteria (AJCC 8 th edition). The following data points were collected: patient demographics (age, sex) and tumor characteristics (location; size; tumor thickness/depth of invasion; invasion of adjacent structures, such as skin, bone, masticator space, and infratemporal fossa). The clinical T stage (cT), pathological T stage (pT), and adjuvant treatment details were collected.

### Statistical Analysis

The concordance between clinical and pathological T staging was analyzed using appropriate statistical tests. Cohen’s kappa coefficient was used to measure the agreement between clinical and pathological T staging. The data were analyzed using SPSS, and a *p*-value of < 0.05 was considered statistically significant.

## Results

### Patient Characteristics

A total of 469 patients with gingivobuccal complex cancers were included in the study. The median age of the patients was 50.8 years (range: 22–82 years); 91.3% were males and 8.7% were females. The majority of the patients (74.7%) had primary tumors located in the buccal mucosa. The demographic and baseline clinical characteristics of the patients are summarized in Table 1.

**Table 1:** The demographic and baseline clinical characteristics of the patients

Characteristic	Value
Total number of patients (*N*)	469
Age (years)	
Median (range)	50.8 years (22–82)
Gender	
Male (%)	91.3%
Female (%)	8.7%
Tumor location	
Buccal mucosa (%)	74.7%
Retromolar trigone (%)	4.8%
Gingivobuccal sulcus (%)	4.1%
Alveolus (%)	17.4%
Tumor grade	
Well differentiated (%)	8.9%
Moderately differentiated (%)	66.8%
Poorly differentiated (%)	24.9%

## Comparison of Clinical and Pathological T Staging

Of the 469 patients, clinical T staging (cT) was performed based on preoperative clinical examination and radiological imaging, while pathological T (pT) was assessed after the surgical resection (Table 2).

**Table 2:** Comparison of clinical and pathological T staging

Clinical T staging distribution (%)		Pathological T staging distribution (%)	
Stage	Number (%)	Stage	Number (%)
cT1	22 (4.7%)	pT1	61 (13%)
cT2	86 (18.3%)	pT2	139 (29.6%)
cT3	25 (5.3%)	pT3	66 (14.1%)
cT4a	114 (24.3%)	pT4a	203 (43.2%)
cT4b	216 (46%)	pT4b	0

## Concordance Between Pathological and Clinical T Staging

The overall concordance rate between clinical and pathological T staging was 32.08%. The detailed comparison is shown in Table 2. Out of 469 patients 6.74% had upstaging, i.e. pT > cT, whereas 34.4% had downstaging (pT < cT). However, the majority of the downstaging was from T4b to T4a (16.2%), which has not resulted in any change in the adjuvant treatment plan; 13.4% patients had upgrading of the staging, whereas 51.4% had downstaging. Majority of patients who had downstaging was due to disagreement in the involvement of masseter muscle and infratemporal fossa between clinical and pathological evaluations. The concordance and discordance between the clinical and pathological T staging are given in Table 3.

**Table 3:** The concordance and discordance between the clinical and pathological T staging

Clinical T stage	Pathological T stage	Number of patients	Percentage (%)	Concordance (%)	Upstaging (%)	Downstaging (%)
T1	T1	7		1.49	-	-
	T2	2		-	0.42%	-
	T3	0		-	0%	-
	T4	0		-	0%	-
T2	T1	21		-	-	4.47%
	**T2**	66		**14.07%**	-	-
	T3	7		-	1.49%	-
	T4	18		-	3.83%	-
T3	T1	4		-	-	0.85%
	T2	6		-	-	1.20%
	T3	12		2.41%	-	
	T4	5		-	1.00%	-
T4 A	T1	4		-	-	0.80%
	T2	8		-	-	1.61%
	T3	8				1.61%
	**T4a**	70		**14.11%**		
T4B	T1	4		-	-	0.85%
	T2	25				5.04%
	T3	17		-	-	3.42%
	**T4a**	76		-	-	**16.2%**
	T4b	0		0%	-	-

## Discrepancies in Tumor Size, Depth of Invasion, Bone, Skin, and Masticator Space Involvement

Tumor size and tumor thickness discrepancies between clinical and pathological assessments were observed in 12.9% of cases.

Bone erosion was clinically suspected or detected in 49.3% of patients based on radiological and clinical examination. However, pathological examination confirmed bone erosion in 28.4% of patients with five patients having superficial cortical bone erosion. The kappa coefficient value was 0.423.

Masseter involvement was found in 48% and ITF involvement in 32% of the patients preoperatively.

Skin involvement was clinically suspected in 19.2% of cases, but pathological findings confirmed skin invasion in 10.9% of cases with a kappa coefficient value of 0.513.

## Discussion

In this retrospective study of 469 patients with gingivobuccal complex (GBC) cancers, we found that the overall concordance rate between clinical and pathological T staging was 32.08%, with significant discrepancies observed in both upstaging and downstaging. While 13.4% of patients experienced upstaging (pT > cT), a much larger proportion, 51.4%, had downstaging (pT < cT). These findings highlight the frequent divergence between clinical assessment and the more definitive pathological evaluation, underscoring the limitations of preoperative clinical and radiological evaluations in accurately staging GBC cancers.

Our results are consistent with the growing body of literature that has reported low concordance rates between clinical and pathological staging across head and neck cancers. In a study by Koch et al., disparity was found in at least one of the tumor parameters in 50% of the cases [2]. Likewise, studies by Choi et al. and Ho et al. noted that discrepancies in tumor size, depth of invasion, and local tissue involvement frequently led to incorrect clinical staging, affecting treatment decisions [3].

One of the most striking findings of our study was the significant discrepancy in tumor size and depth of invasion between clinical and pathological evaluations. Discrepancies were observed in 12.9% of cases, with Kruskal–Wallis rank-sum test revealing a statistically significant difference between clinical and pathological tumor size measurements (*p* = 0.003). Similar findings have been reported in previous studies on OSCC. The clinical tumor size was often overestimated, especially in cases where tumors involved complex anatomical regions like the buccal mucosa and retromolar trigone. This overestimation can lead to overaggressive surgical approaches, highlighting the need for more precise imaging or intraoperative assessments [4].

Depth of invasion (DOI) has emerged as a critical factor in the prognosis and staging of oral cancers, as demonstrated by recent updates to the AJCC Cancer Staging Manual (8 th edition) [5]. A study by Ebrahimi et al. showed that DOI is a strong predictor of nodal metastasis and survival, emphasizing the importance of accurate preoperative assessment [6]. Our findings suggest that clinical assessments may underestimate DOI in a significant number of cases, potentially underestimating the aggressiveness of the tumor and leading to insufficient treatment.

### Discrepancies in Bone Involvement

Bone involvement is a key factor in determining T4 staging in GBC cancers, yet our study found significant discrepancies in its clinical detection. Bone erosion was clinically suspected in 49.3% of cases but confirmed pathologically in only 28.4%. Previous studies have reported similar findings, with radiological assessments often overestimating bone involvement due to limitations in CT and MRI resolution [7].

The distinction between superficial cortical bone erosion and full-thickness involvement is clinically significant, as it can influence the extent of surgical resection and the need for adjuvant therapy. Our findings suggest that clinical overestimation of bone involvement may lead to more radical surgeries than necessary. Advanced imaging techniques, such as high-resolution MRI or PET-CT, may offer better precision in detecting bone invasion and should be considered in complex cases where clinical and radiological findings are ambiguous [8].

### Discrepancies in Masticator Space, Infratemporal Fossa, and Skin Involvement

Involvement of the masticator space and infratemporal fossa plays a crucial role in defining T4b stage, indicating unresectable disease. Our study found that masticator space involvement was clinically suspected in 48% of patients and infratemporal fossa involvement in 32%; however, pathological confirmation was inconsistent. This discrepancy underscores the challenges in evaluating deep tissue invasion in the preoperative setting. As per literature, MRI and CT often lack the resolution to distinguish between actual tumor invasion and inflammatory or reactive changes in these complex anatomical spaces, such as masticator space and infratemporal fossa [9]. In our results, majority of the downstaging was due to involvement of these complex spaces which is T4b. However, downstaging from T4b to T4a will not make any difference in the adjuvant treatment plan.

In addition, skin involvement was clinically suspected in 19.2% of cases but confirmed pathologically in only 10.9%. The overestimation of skin involvement can occur in background of inflammation or secondary skin changes, which were often mistaken for direct tumor invasion [10]. These findings suggest that reliance on clinical judgment alone can lead to inaccurate staging and potentially overtreatment.

Pathological staging should therefore be integrated into the final treatment decision-making process, particularly in cases where clinical and radiological findings are ambiguous or inconsistent. Multimodal imaging approaches, including PET-CT and high-resolution MRI, may help improve the accuracy of clinical staging in the future.

One of the key concerns arising from this study is the group of patients who experienced downstaging from T4 or T3 to T2 or T1, which ultimately excluded them from receiving adjuvant treatment. Out of the 469 patients, 56 were downstaged and did not receive adjuvant therapy. Notably, 21 of these patients were downstaged from T4b to either T2 or T1. This highlights the inherent challenges in accurately diagnosing T4b disease through pathological assessment. In such cases, adjuvant treatment decisions should be guided by clinicoradiological staging rather than solely relying on pathological findings.

## Conclusion

In conclusion, this study demonstrates significant discrepancies between clinical and pathological T staging in gingivobuccal complex cancers, with a concordance rate of only 32.08%. The high rate of downstaging, particularly from cT4b to pT4a, suggests that clinical staging often overestimates the extent of disease. We recommend better histopathological examination of T4b disease by tagging or marking each structure of masticator or infratemporal fossa separately, which will help in better treatment decisions.

## Data Availability

Data were collected retropsectively from the hospital electronic medical records.
